# A Rare Case of Community-Acquired Acinetobacter Urethritis in an Immunocompetent Adult Woman

**DOI:** 10.7759/cureus.115617

**Published:** 2026-09-01

**Authors:** Alejandro Arbona-Lampaya, Ricardo G Blanco-Ortiz, Jan P Villanueva, Roberto Angeli-Morales, Diego Girald-Rodriguez, Allen Roman, Radames Roman-Grau

**Affiliations:** 1 School of Medicine, University of Puerto Rico, Medical Sciences Campus, San Juan, PRI; 2 Department of Obstetrics and Gynecology, University of Puerto Rico, Medical Sciences Campus, San Juan, PRI

**Keywords:** acinetobacter, community-acquired urinary tract infection, sulfamethoxazole, trimethoprim, urethritis

## Abstract

Urethritis is most commonly caused by sexually transmitted pathogens; however, refractory presentations may indicate an atypical etiology that does not respond to empiric therapy. *Acinetobacter* species are Gram-negative bacilli classically associated with nosocomial infections and are an uncommon cause of community-acquired lower urinary tract infections in immunocompetent women. We present a case of severe community-acquired urethritis caused by *Acinetobacter baumannii* complex in a previously healthy 44-year-old woman, with concurrent isolation of *Acinetobacter lwoffii* of uncertain pathogenic significance. The patient presented with dysuria and pelvic pain that persisted despite a single 500-mg intramuscular dose of ceftriaxone. Nucleic acid amplification testing was negative for *Chlamydia trachomatis* and *Neisseria gonorrhoeae*. Urinalysis demonstrated microscopic hematuria without pyuria, and urine cultures showed no bacterial growth. Urethral swab culture demonstrated moderate growth of both organisms, with identification and antimicrobial susceptibility testing performed using the VITEK 2 automated system. Both isolates were susceptible to trimethoprim-sulfamethoxazole (TMP-SMX), and a 10-day course resulted in complete resolution of symptoms. This case expands the recognized clinical spectrum of *Acinetobacter* infections by demonstrating their potential to cause community-acquired urethritis in immunocompetent women. It highlights the diagnostic value of urethral swab culture in patients with refractory lower urinary tract symptoms and negative urine cultures and proposes a stepwise clinical framework for evaluating urethritis that fails to respond to empiric therapy.

## Introduction

Urethritis, defined as inflammation of the urethra, may result from infectious or non-infectious etiologies, with the most common infectious causes being *Chlamydia trachomatis* and *Neisseria gonorrhoeae*, and less frequently *Trichomonas vaginalis* [1,2]. Most female patients are asymptomatic; however, when symptoms are present, dysuria is the most common manifestation [3]. The majority of *Acinetobacter* spp. infections are nosocomial and most frequently involve the respiratory tract, particularly in cases of ventilator-associated pneumonia, as their pathogenicity is often associated with biofilm formation on medical devices, including endotracheal tubes and catheters [4,5]. Urinary tract infections caused by *Acinetobacter* spp. predominantly occur in men and are most often acquired in healthcare settings [6]. Among female patients, reported cases generally involve nosocomial infections and are frequently associated with drug resistance [5-7]. Mild, self-limiting cases of cystitis or other lower urinary tract infections have also been reported [7]. Here, we present the first documented case of severe, community-acquired urethritis caused by *Acinetobacter* *baumannii *complex, with concurrent isolation of *Acinetobacter lwoffii*.

## Case presentation

A previously healthy 44-year-old woman from Puerto Rico presented to an outpatient clinic with a two-week history of progressively worsening lower urinary tract symptoms. Her symptoms initially consisted of severe dysuria and pelvic pain, for which she sought evaluation in an emergency department. Renal ultrasonography performed during that visit demonstrated a renal calculus, which passed spontaneously without intervention. She was empirically treated with a single 500-mg intramuscular dose of ceftriaxone for a presumed urinary tract infection and subsequently discharged. Despite antibiotic treatment, her symptoms persisted, and she returned to the outpatient clinic six days later for further evaluation.

On examination, the patient was afebrile and hemodynamically stable. Pelvic examination revealed an enlarged, erythematous urethra that was severely tender to palpation. There was no vaginal discharge or suprapubic tenderness. Urinalysis demonstrated microscopic hematuria without pyuria or nitrites. Urine cultures were negative for bacterial growth. Nucleic acid amplification testing performed at the outpatient clinic was negative for *Chlamydia trachomatis* and *Neisseria gonorrhoeae*. Urethral swab cultures, however, grew *A. baumannii* complex and *A. lwoffii *in moderate growth. Identification and antimicrobial susceptibility testing were performed using the VITEK 2 automated system (bioMérieux, Marcy-l'Étoile, France), and the diagnosis of urethritis was established on the basis of clinical presentation and microbiological findings. Both isolates were susceptible to ampicillin/sulbactam, ceftazidime, gentamicin, piperacillin/tazobactam, and trimethoprim-sulfamethoxazole (TMP-SMX) (Table 1).

**Table 1:** Antimicrobial susceptibility profile of the urethral isolates obtained. Urethral culture demonstrated moderate growth of* Acinetobacter lwoffii *and *Acinetobacter baumannii *complex, both susceptible to multiple antimicrobial agents. MIC: Minimum inhibitory concentration

Organism	Quantity	Antibiotic	Interpretation	MIC (µg/mL)
Acinetobacter lwoffii	Moderate growth	Ampicillin/Sulbactam	Susceptible	≤2
		Ceftazidime	Susceptible	≤0.5
		Gentamicin	Susceptible	≤1
		Piperacillin-Tazobactam	-	-
		Trimethoprim-Sulfamethoxazole	Susceptible	≤20
*Acinetobacter baumannii*complex	Moderate growth	Ampicillin/Sulbactam	Susceptible	≤2
		Ceftazidime	Susceptible	8
		Gentamicin	Susceptible	≤1
		Piperacillin-Tazobactam	Susceptible	16
		Trimethoprim-Sulfamethoxazole	Susceptible	≤20

Upon further questioning, the patient denied recent hospitalization, bladder catheterization, pelvic instrumentation, or prior antibiotic exposure other than the recent course of ceftriaxone. She had no history of recurrent urinary tract infections, sexually transmitted infections, or immunocompromising conditions. She reported being sexually active exclusively with her husband and denied any high-risk sexual contacts. She also reported no recent travel or known exposure to sick contacts.

Based on the antimicrobial susceptibility profile and the outpatient clinical setting, oral TMP-SMX (160 mg/800 mg) twice daily was initiated for a 10-day course. At follow-up on day 10 of treatment, the patient reported marked improvement in dysuria and urinary frequency, with complete resolution of symptoms. She reported no adverse effects related to therapy.

## Discussion

*Acinetobacter *spp. are opportunistic Gram-negative bacilli that have historically been associated with nosocomial infections, particularly among critically ill and immunocompromised patients, largely due to their ability to form biofilms [4,5,8,9]. In addition, these organisms are well recognized for their propensity to acquire antimicrobial resistance, contributing to their emergence as important pathogens in ventilator-associated pneumonia, catheter-related infections, and bacteremia, particularly among patients with prolonged hospital stays [4,5,10].

Community-acquired infections by *Acinetobacter* spp. remain rare, especially those affecting the lower urinary tract in immunocompetent women [7,11]. This species maintains environmental reservoirs in soil, water, and human skin commensal flora, suggesting that inoculation may occur through microtrauma, hygiene practices, or direct environmental contact [7,9,11]. The precise route of exposure in community-based settings remains poorly characterized and warrants further epidemiological investigation.

To our knowledge, this case represents the first documented instance of community-acquired urethritis caused by *A. baumannii* complex in an otherwise healthy woman, with concurrent isolation of *A. lwoffii*. Previous reports have predominantly described hospitalized or catheterized patients, in whom infection typically develops following colonization of an indwelling device or prolonged antibiotic exposure [4,5,6,10]. *A. baumannii *complex is characterized by multidrug resistance and a strong association with nosocomial environments [4,5,10]. The recovery of *A. baumannii *complex from a site-specific urethral specimen in a patient without traditional risk factors, including no history of hospitalization, catheterization, or immunosuppression, highlights its potential to cause community-acquired disease and supports consideration of *Acinetobacter* as a potential uropathogen even in otherwise healthy women. *A. lwoffii*, which is commonly found on the skin and mucosal surfaces, has also been associated with sporadic infections in immunocompetent hosts [7]. However, given its recognized commensal presence on skin and mucosal surfaces, its contribution to the patient's clinical presentation cannot be definitively determined based on culture findings alone and is therefore considered a concurrent isolate of uncertain clinical significance. Additional microbiological evidence, such as point-of-care Gram staining of urethral discharge or molecular characterization of virulence factors, was not obtained and would have been necessary to establish dual pathogenicity.

From a diagnostic perspective, this case underscores the limitations of urine cultures for detecting atypical urethral pathogens [2]. Despite the presence of severe dysuria, hematuria, and an erythematous urethra, all findings consistent with active urethritis, the urine culture remained negative. Although the concomitant renal calculus may have contributed to the hematuria, the absence of pyuria on urinalysis is compatible with urethritis, in which inflammation is confined to the urethral mucosa and may not generate sufficient leukocytes in voided urine to meet the criteria for pyuria. The patient's prior ceftriaxone exposure at the time of urethral swab collection likely contributed to the negative urine culture. Notably, *A. baumannii *complex exhibits intrinsic resistance to cephalosporins, which may explain its recovery from the urethral swab despite prior ceftriaxone exposure and further supports its role as the causative pathogen. Urethral swab culture, which is recommended as part of the standard diagnostic evaluation for recurrent or refractory urethritis [2], yielded moderate growth of both organisms and established the diagnosis, emphasizing the clinical utility of site-specific sampling in patients with persistent lower urinary tract symptoms despite empiric therapy (Figure 1).

**Figure 1:**
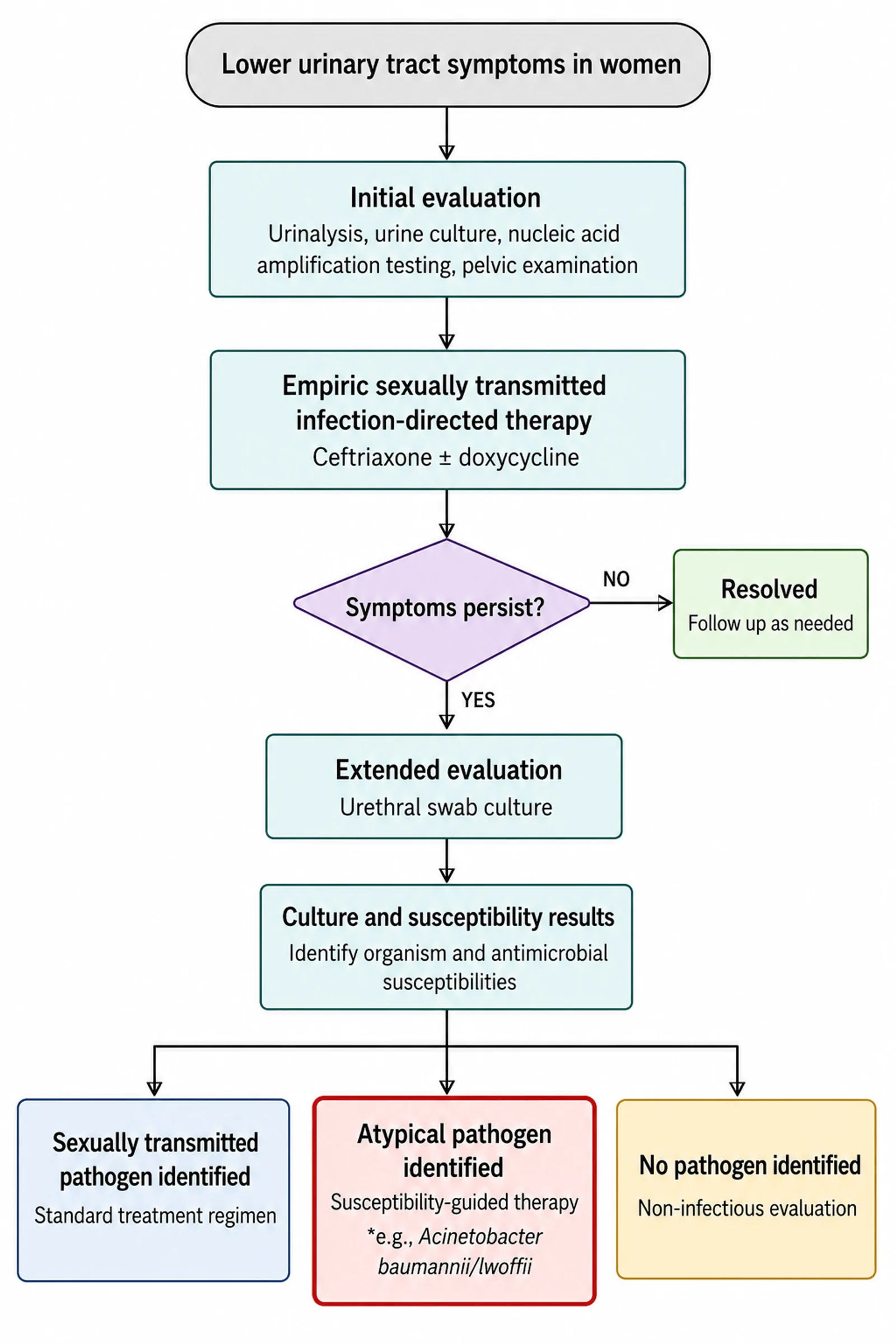
Proposed diagnostic algorithm for the evaluation of refractory urethritis in women. Following initial evaluation and empiric sexually transmitted infection-directed therapy, persistent symptoms should prompt extended evaluation with urethral swab culture. Culture and susceptibility results guide definitive management across three pathways: sexually transmitted pathogen identified, atypical pathogen identified, or no pathogen found. The center pathway reflects the index case. *Acinetobacter baumannii complex and *Acinetobacter lwoffii*. Algorithm developed based on CDC STI Treatment Guidelines [1] and Sell et al. [2] Figure 1 was created with the assistance of Claude AI (claude-sonnet-4-6; Anthropic, San Francisco, CA, United States).

Empiric treatment of urethritis in women is generally directed against common sexually transmitted pathogens, including *Chlamydia trachomatis*, *Neisseria gonorrhoeae*, and *Trichomonas vaginalis*, in accordance with CDC guidelines [1,2]. This standardized treatment strategy may delay recognition of atypical pathogens, particularly when their clinical presentations overlap substantially with those of more common etiologies. In this case, nucleic acid amplification testing was negative for *C. trachomatis* and *N. gonorrhoeae*. Although testing for *T. vaginalis* was not performed, active trichomonal infection is considered unlikely given the patient's complete clinical response to TMP-SMX, which has no antitrichomonal activity. The persistence of symptoms despite prior ceftriaxone therapy nevertheless prompted further diagnostic evaluation, ultimately leading to identification of the causative pathogen.

Based on the diagnostic approach used in this case and existing guideline recommendations, we propose a stepwise clinical framework for evaluating women with refractory urethritis (Figure 1). This algorithm was developed using established guideline principles and the current case and should not be considered an evidence-based recommendation until validated in larger clinical series. Initial evaluation consists of urinalysis, urine culture, nucleic acid amplification testing, and pelvic examination, followed by empiric therapy targeting* N. gonorrhoeae* and *C. trachomatis* in accordance with CDC guidelines [1]. Resolution of symptoms at this stage warrants routine follow-up. For patients with persistent symptoms, further evaluation with urethral swab culture is recommended, consistent with guidance supporting site-specific sampling in recurrent or refractory presentations [2]. This step is particularly important because standard urine culture may fail to detect atypical urethral pathogens, as demonstrated in the present case [2]. Subsequent management is guided by culture and antimicrobial susceptibility results and follows one of three pathways: identification of a sexually transmitted pathogen requiring treatment with a standard regimen, identification of an atypical pathogen requiring susceptibility-guided therapy, or a negative culture prompting evaluation for non-infectious etiologies. The present case followed the second pathway, with urethral swab culture identifying *A. baumannii* complex as the primary causative pathogen, thereby guiding targeted oral TMP-SMX therapy and resulting in complete resolution of symptoms.

Management of *Acinetobacter* infection can be challenging given the species' acquired resistance mechanisms [4,5,10]. However, the isolates in the present case demonstrated full susceptibility to multiple agents, including TMP-SMX, consistent with a community-acquired strain differing from the multidrug-resistant profiles typical of nosocomial counterparts [5,10]. Oral TMP-SMX therapy was effective, resulting in complete resolution of symptoms.

Several limitations of this case should be acknowledged. Organism identification was performed using the VITEK 2 automated system, which identifies *A. baumannii* at the complex level and does not permit definitive species-level differentiation within the complex; molecular confirmation using 16S rRNA gene sequencing was not performed. The pathogenic contribution of *A. lwoffii* cannot be determined based on culture findings alone given its recognized commensal status. Additionally, point-of-care Gram staining of urethral discharge was not performed at the time of clinical evaluation, which may have provided earlier evidence of inflammation and aided in diagnosis. Testing for *T. vaginalis* was not performed, although active trichomonal infection is considered unlikely for the reasons described above. Finally, the proposed diagnostic algorithm is derived from a single case and established guideline principles and should therefore be regarded as a proposed clinical framework rather than an evidence-based recommendation.

## Conclusions

This case highlights several important clinical considerations. First, *A. baumannii *complex should be included in the differential diagnosis of refractory urethritis, particularly when symptoms persist despite empiric therapy directed against sexually transmitted infections. Second, a negative urine culture in the presence of clinical features characteristic of urethritis does not exclude infection, and targeted urethral swab culture should be considered in refractory presentations. Third, community-acquired *Acinetobacter* infections may demonstrate favorable antimicrobial susceptibility patterns, allowing treatment with oral agents. Finally, this case emphasizes the need for clinicians to recognize atypical uropathogens in immunocompetent patients and the importance of site-specific microbiological sampling for establishing the diagnosis. The concurrent isolation of *A. lwoffii *further illustrates the challenge of distinguishing true co-infection from commensal colonization in urethral specimens, and future cases should incorporate point-of-care Gram staining and molecular characterization to help clarify this distinction.

## References

[1] Workowski KA, Bachmann LH, Chan PA (2021). Sexually Transmitted Infections Treatment Guidelines, 2021. MMWR Recomm Rep.

[2] Sell J, Nasir M, Courchesne C (2021). Urethritis: rapid evidence review. Am Fam Physician.

[3] Tuddenham S, Hamill MM, Ghanem KG (2022). Diagnosis and treatment of sexually transmitted infections: a review. JAMA.

[4] Maragakis LL, Perl TM (2008). Acinetobacter baumannii: epidemiology, antimicrobial resistance, and treatment options. Clin Infect Dis.

[5] Cavallo I, Oliva A, Pages R (2023). Acinetobacter baumannii in the critically ill: complex infections get complicated. Front Microbiol.

[6] Djordjevic Z, Folic MM, Zivic Z, Markovic V, Jankovic SM (2013). Nosocomial urinary tract infections caused by Pseudomonas aeruginosa and Acinetobacter species: sensitivity to antibiotics and risk factors. Am J Infect Control.

[7] Wong D, Nielsen TB, Bonomo RA, Pantapalangkoor P, Luna B, Spellberg B (2017). Clinical and pathophysiological overview of Acinetobacter infections: a century of challenges. Clin Microbiol Rev.

[8] Gedefie A, Alemayehu E, Mohammed O, Bambo GM, Kebede SS, Kebede B (2023). Prevalence of biofilm producing Acinetobacter baumannii clinical isolates: a systematic review and meta-analysis. PLoS One.

[9] Joly-Guillou ML (2005). Clinical impact and pathogenicity of Acinetobacter. Clin Microbiol Infect.

[10] Sunenshine RH, Wright MO, Maragakis LL (2007). Multidrug-resistant Acinetobacter infection mortality rate and length of hospitalization. Emerg Infect Dis.

[11] Munoz-Price LS, Weinstein RA (2008). Acinetobacter infection. N Engl J Med.

